# Supplementary material for: STAT3 Signalling Drives LDH Up-Regulation and Adiponectin Down-Regulation in Cachectic Adipocytes
Source: Int J Mol Sci. 2023 Nov 15;24(22):16343. doi: 10.3390/ijms242216343 (PMC10671608; doi:10.3390/ijms242216343)
Supplement: Supplementary file 1 [file ijms-24-16343-s001.zip › ijms-2691476-supplementary.pdf]

Supplementary Figure S1

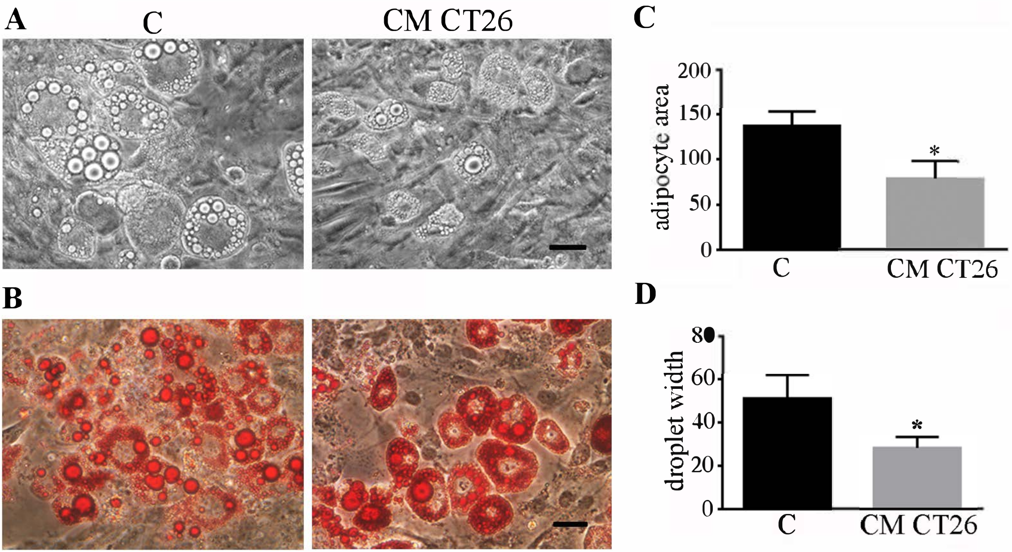

The treatment of murine adipocytes with CM CT26 induces cachectic features. Adipocytes were treated with CM CT26 for 48 hours. A) Representative images of control (C) and CM CT26-treated adipocytes; B) Red oil staining; C) Area of adipocytes; D) Width of lipid droplets. The measures showed in C) and D) are obtained measuring adipocyte area and lipid droplets with Image J in at least ten randomly chosen fields. C, control adipocytes; *$p<0.05$; $n = 4$; scale: 50 µm.

Red oil staining: after the treatment cells were washed with PBS and then fixed with 4% para-formaldehyde for 30 minutes at 4° C. Cells were treated with 60% isopropanol for 5 minutes and then with Oil Red working solution (#01391, Sigma-Aldrich) (3-part Oil Red: 2-part water) for 15 minutes at room temperature. After three washes with water, cells were photographed under the optical microscope.

Supplementary Figure S2

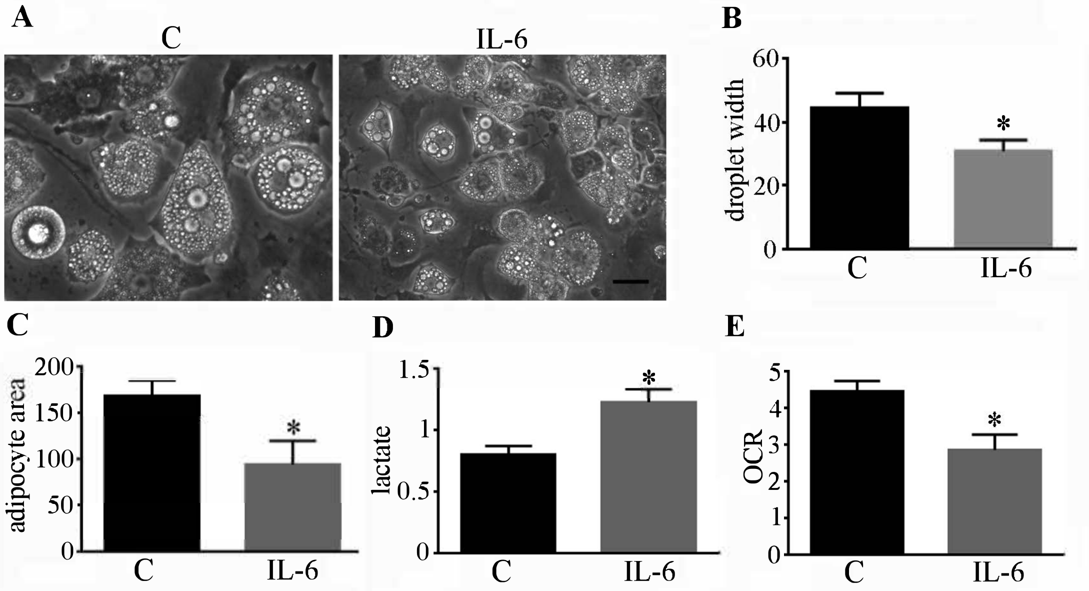

IL-6 induces cachectic features in murine adipocytes. Adipocytes were treated with IL-6 (240 ng/ml) for 48 hours. A) Representative images of control (C) and IL-6-treated adipocytes; B) Width of lipid droplets; C) Adipocytes' area. The measures showed in B) and C) are obtained with ImageJ using at least ten randomly chosen fields. D) Lactate amount; E) Oxygen Consumption Rate (OCR). C, control adipocytes; *p<0.05; n = 4; scale: 50 μm.

Supplementary Figure S3

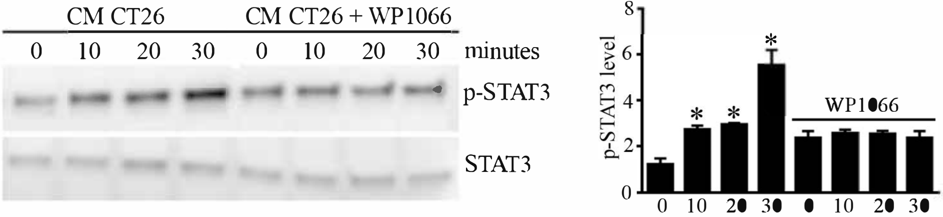

STAT3 inhibitor WP1066 impedes STAT3 phosphorylation by CM CT26. Murine adipocytes were treated with serum-free medium overnight. Cells were pre-treated with WP1066 (10 μm) for one hour and then stimulated with CM CT26 for the indicated times. The level of total and phosphorylated-Tyr705 of STAT3 was detected by immunoblot. The level of total STAT3 has used for normalization and the mean values are reported in the bar graph. *$p<0.05$; $n = 3$
